# Falling behind whom? Economic geographies of right-wing populism in Europe

**DOI:** 10.1080/13501763.2023.2278647

**Published:** 2023-11-09

**Authors:** Dominik Schraff, Jonas Pontusson

**Affiliations:** a Department of Politics and Society, Aalborg University, Aalborg Ø, Denmark; b Department of Political Science and International Relations, University of Geneva, Genève, Switzerland

**Keywords:** Europe, geography, inequality, right-wing populism

## Abstract

Existing studies suggest that right-wing populist parties (RWPPs) appeal to people in communities that have fallen behind in material terms. However, it remains open which benchmark communities apply as they become politically discontented. We argue that the structure of territorial inequalities influences the benchmarks used by people in regions falling behind. Panel data regressions using subnational election results in EU states from 1990 to 2018 reveal a sharp contrast between the economic geographies of right-wing populism in core and peripheral EU member states. We find a strong association between falling behind the richest region of the country and RWPP support within core EU countries, while in peripheral EU states falling behind the EU core is associated with regional support for RWPPs. This suggests that RWPP voters in peripheral countries cue on how they are faring relative to the EU core, while RWPP supporters in core countries cue on how they are faring relative to dynamic regions of their own country. Our analysis also shows that increased manufacturing employment reinforces the effect of falling behind the richest region in core EU member states, while we find no strong evidence that regional economic stagnation is important to the electoral performance of RWPPs.

This article explores economic determinants of electoral support for right-wing populist parties at the subnational (regional) level in the European Union from 1990 to 2018. Building on existing literature, we focus on the rise of territorial inequalities as a source of the resentment that apparently motivates many right-wing populist voters. It is a commonplace to observe that support for right-wing populist parties is geographically concentrated and that regions that have fallen behind economically are the bastions of right-wing populist support. To cite just one recent example, the Sweden Democrats won 20.5 per cent of the national vote in 2022, making them the country’s largest non-Left party, but they only won 10.5 per cent of the vote in Stockholm, the country’s largest city and the electoral district with the fastest economic growth since the previous election.^1^ The implication of the ‘falling-behind literature’ would seem to be that right-wing populist support should be weaker in countries where regional disparities have not grown so much. As we shall document in what follows, there is an important contrast between the Northwest core and the Southern and Eastern periphery of the European Union in this respect. Overall, the core has experienced more rapid and more ‘knowledge-intensive’ growth than the periphery since the financial crisis of 2007–2008, but it has also (perhaps for that very reason) experienced a much more dramatic increase of regional disparities. Yet it is evidently not the case that right-wing populism thrives in the core alone (witness recent elections in Slovakia, Spain, Hungary, Poland, and Italy). We argue that this apparent puzzle can at least partly be solved by recognising that perceptions of falling behind can be based on cross-national as well as national benchmarks. Crudely put, right-wing populists in the core resent the richer regions in their country while right-wing populists in the periphery resent richer countries in the European Union.

Having established the that different forms of territorial inequality matter in the core and periphery of the European Union, we proceed to explore two hypotheses that pertain to conditioning of the effects of territorial inequalities on regional support for right-wing populist parties. The first hypothesis posits that vertical inequality distracts from horizonal inequality or, in other words, that the right-wing populist response to falling behind other regions or countries will be most pronounced in regions characterised by a relatively equal distribution of income. The second hypothesis is that regional economic growth moderates the right-wing populist response to falling behind other regions or countries. To anticipate, we find some support for the first hypothesis and surprisingly little support for the second.

Our analysis leverages a new dataset on parliamentary election results at the sub-national level, the European NUTS-Level Election Dataset (EU-NED). Encompassing 1,195 regional units, the EU-NED records party vote shares at the lowest regional level available (NUTS 2 or 3) for EU member states and associated countries from 1990 to 2018 (Schraff et al., 2023).^2^ Matching these data with EU data on GDP per capita for the same regional units, we estimate the direct effects of relative as well as absolute economic performance on support for right-wing populist parties as well as the effects of interactions of these variable. For reasons to which we shall return, we treat the manufacturing share of regional employment as a proxy for within-region income inequality. Again, we estimate the direct effects of this variable as well as the effects of interacting it with our measures of relative economic performance. Importantly, the models that we rely on to estimate these effects are two-way fixed effects models (with dummies for years and regions). We thus identify *the effects of changes in economic conditions on regional support for right-wing populist parties*.

The rest of the article is organized as follows. First, we elaborate on our understanding of right-wing populism, discuss alternative benchmarks for social comparisons and develop our core hypotheses regarding the effects of changes in absolute as well as relative income and the effects of changes in manufacturing employment. Secondly, we provide further information about the data we analyze and present descriptive data patterns. Thirdly, we specify the models that we estimate and introduce control variables. Fourthly, we present the empirical results. Finally, we discuss the implications of our results, point out the limitations of our analysis and suggest avenues for further theorising and research.

## 1. Literature and theory

The dependent variable of our empirical analysis is the regional vote share of right-wing populist parties in elections to national parliaments. We operationalise ‘right-wing populist parties’ (henceforth RWPPs) by combining the PopuList coding of parties as ‘populist’ and as ‘far Right’ (Rooduijn et al., 2019). Following Mudde (2004, 2007), the PopuList coding scheme defines ‘populist parties’ as parties that argue that society is separated into two homogeneous and antagonistic groups – the (pure) people versus the (corrupt) elite – and claim to represent the general will of the people. ‘Far Right parties’ are in turn defined as nativist and authoritarian.^3^

The rise of RWPPs (and right-wing populist forces within mainstream right parties) has attracted a lot of scholarly attention in recent years. Most prominently, Inglehart and Norris (2019) identify cultural values related to immigration, cosmopolitanism and supra-national governance as the key determinants of individual support for right-wing populist parties and argue that the rise of right-wing populism should first and foremost be seen as a reaction against the cultural and political advances of the postmaterialist Left since the 1980s. Against this ‘culturalist’ interpretation, a number of recent studies seek to explain right-wing populism as a response to some combination of technological change, rising income inequality and economic stagnation. Such ‘materialist’ alternatives to Inglehart and Norris’ account take essentially two forms.

Focusing on inequalities between individuals or households, a number of studies show that the effects of labor-market insecurity and relative deprivation are not linear (Burgoon et al., 2019; Engler & Weisstanner, 2021; Kurer, 2020; Rovny & Rovny, 2017). Crudely put, the core message of these studies is that right-wing populist parties appeal to people in the lower middle of the income distribution who have fallen behind or, at least, perceive themselves as having fallen behind in terms of social status and relative income (e.g., self-employed, clerical workers, semi-skilled and skilled production workers). The populist voters are not the poorest and most precarious segment of the electorate; rather, they are people who think they used to be part of the ‘prosperous middle class’ and no longer see themselves as such (see also Gidron & Hall, 2019).

The other alternative to Inglehart and Norris’ account of the rise of right-wing populism shifts the focus of attention from individuals to communities or other territorial units. From this perspective, right-wing populism appeals to a broad spectrum of people in communities that have fallen behind or see themselves as being at risk of falling behind. Again, social-status decline as well as relative-income loss feature as motivations of right-wing populist voters in this literature.^4^ The empirical analysis presented in this paper pertains to the literature on the relationship between territorial inequalities and right-wing populism, but we seek to make a broader contribution by bringing to the fore the question of benchmarks or, in other words, ‘reference groups.’ The notion of ‘falling behind’ occupies a central place in both alternatives to Inglehart and Norris’ account, yet this literature often seems to skirt, or take for granted, the obvious question: Falling behind whom? Put differently, what are the social comparisons behind the discontent that motivates right-wing populist voters across Europe? A definitive answer would require a customised survey, perhaps with an experimental design, but we believe that insights into this question might also be gained through an analysis of regional variation in support for right-wing populist parties.

There is broad consensus in existing literature that resentment and anger are important motivations for many people who are attracted to right-wing populist rhetoric and programmes (Gonthier, 2023). While Cramer (2016) provides a penetrating analysis of rural resentment in Wisconsin, Gest (2016) documents the resentment of middle-age male workers living in American and British towns that have experienced de-industrialization over several decades. In a different vein, Burgoon et al. (2019) emphasise that ‘scapegoating’ features prominently in the appeal of RWPPs: scapegoating of immigrants, racial minorities and, more generally, undeserving recipients of welfare benefits or other kinds of government favors. Crucially, we think, the rhetoric of right-wing populists combines this focus on the undeserving beneficiaries of public policy with a critique of political elites – the national political establishment (‘Washington’ in the case of the US), but also supranational elites (Brussels).^5^ In the world view of Trump voters and their European equivalents, it is not Blacks or immigrants that are to be blamed, but rather free-trade and socially-liberal elites who favor Blacks and immigrants over ‘ordinary people.’^6^

As noted by aforementioned scholars, and many others (see Ejrnaes et al., 2023), the politics of resentment appear to be closely linked to perceptions of relative deprivation or, more precisely, ‘falling behind’ in terms of income, opportunities, and social status. For present purposes, it is useful to recall to Runciman’s classic (1966) discussion of relative deprivation. Following Runciman, subjective perceptions of relative deprivation depend not only on identifying oneself as a member of some group or community based on ‘linked fate’, but also, on comparing one’s situation to that of some reference group (to which one does not belong). In Runciman’s assessment, resentment of class inequalities was relatively limited in postwar Britain because most individuals, especially manual workers, tended to adopt highly restrictive reference groups, comparing themselves to people more or less like themselves (e.g., workers in other sectors or workers with other skill profiles).

‘Falling behind’ – or ‘becoming relatively deprived’ – might be conceived in terms of gender, occupations, sectors or racial/ethnic groups, but it can also be conceived in territorial terms, i.e., as one’s own community falling behind other communities, as one’s region falling behind other regions or, perhaps, as one’s country falling behind other countries. Territorial conceptions of societal cleavages arguably make for a better fit with the populist idea of politics as a struggle between ‘ordinary people’ and ‘elites’ than vertical conceptions since the latter, by definition, direct attention to conflicts among people who live or work in proximity with each other. Put differently, right-wing populists (voters as well as parties) are keen to focus attention on inequalities and conflicts between rather than within territorially defined communities. By the same token, we hypothesise that right-wing populist ideas have particular traction in communities that are relatively equal and fall behind other communities. On the other side of the political spectrum, Left parties (and their core voters) arguably find it difficult to address territorial inequalities because of their traditional emphasis on class inequality, making them more inclined to mobilise in favor of women and disadvantaged minorities than in favor of poor regions.

Especially in the context of the European Union, the benchmarking that provides the basis for territorially based perceptions of falling behind plausibly involves cross-national comparisons. Indeed, the preferred option of populist parties with a nativist/nationalist orientation must be to mobilise resentment against privileged people outside the national borders. Framing the ‘problem of territorial inequality’ in this manner maximises the mobilizational potential as well as the ideological coherence of RWPPs. However, there are obvious limits to such strategy in the rich member states that constitute the core of the EU. In these countries, RWPPs may mobilise against the transfer of sovereignty to the EU on principled (political) grounds, but they cannot credibly claim that European integration has benefitted other countries at the expense of their country. By contrast, resentment of the rich EU core would seem to be a very viable basis for nationalist-populist mobilisation in the crisis-hit member states of Southern Europe as well as the new (formerly state-socialist) member states of Eastern Europe. Consistent with this line of reasoning, Santana et al. (2020) argue persuasively that opposition to ‘Europe,’ not just the EU, is a more important determinant of right-wing populist support in Eastern Europe than in Western Europe.^7^

Another crucial difference between the core and the periphery of the European Union pertains to within-country regional economic disparities. As we shall document below, the core EU member states are characterised by much greater inter-regional economic disparities than the peripheral EU member states. Moreover, inter-regional disparities in the core member states grew significantly from the mid-2000s to the late 2010s while inter-regional disparities in the peripheral member states were essentially stable over this period. If territorially based resentment motivates RWPP voters in the core EU member states, this is likely to be resentment of richer regions in their own country rather than richer countries in the EU.^8^

There are multiple ways to measure inequality between regions (see McCann, 2020). From the point of view of the literature that relies on the notion of falling behind, a particularly relevant measure, already employed by Lipps and Schraff (2021), is distance to the country’s richest region. In the empirical analysis that follows, we rely on GDP per capita to measure distance to the richest region and estimate the effects of (changes in) the distance to the richest region on (changes in) the RWPP vote share. Following from the preceding discussion, we expect distance to the richest region to be a better predictor of the regional RWPP vote share in the EU core than in the EU periphery. We also test the hypothesis that distance to the average GDP per capita of member states that we code as ‘core EU member states’ (see below) is a significant predictor of regional RWPP vote share in peripheral member states.

The key idea behind these hypotheses is that that there is more than one social comparison – or benchmark – that might generate populist resentment and that the salience of different benchmarks is context-dependent, varying across countries (and perhaps across regions as well). In a similar vein, Aytac’s (2017) approach to economic voting identifies two different benchmarks that voters may use to evaluate incumbents – other countries’ performance over the same time period or the country’s own performance in a previous period – and argues that the salience of cross-national benchmarking relative to ‘within-country temporal benchmarking’ (our expression) is a function of the country’s level of education and trade exposure. Similarly, we posit that objective conditions matter, but allow for the possibility that political actors may be able to focus public attention on one benchmark rather than another.^9^

The idea that salient benchmarks vary across ‘macro contexts’ distinguishes our approach from that of survey-based studies of RWPP support that take social comparisons into account. For example, Burgoon et al. (2019) posit that slow income growth relative to the poor lead people in the middle to support RWPPs while slow income growth relative to the rich lead people in the middle to support left-wing populist parties. In their theoretical framework and empirical analysis, it is unclear whether people are more responsive to one or the other ‘register’ of relative income growth and whether the degree of ‘unequal responsiveness’ varies across countries or over time. Assuming that political behaviour is simply (universally) a function of relative income growth, the framework presented by Burgoon et al. (2019) implies that rising household inequality, especially (but not only) high-end inequality, benefits left-wing populist parties relative to right-wing populist parties. It seems difficult to reconcile this implication with the strong right-wing tilt of the ‘populist phenomenon’ in most European countries.

Going beyond the core hypotheses set out above, our analysis explores two hypotheses that pertain to conditioning of the political effects of falling behind the richest region or the EU core. The first of these subsidiary hypotheses pertains to within-region inequality. Seeking to correct the exclusive focus on inter-regional inequalities among economic geographers, Lenzi and Perucca (2021) argue that that intra – and inter-regional inequality both generate political discontent. Their mutual reinforcement hypothesis makes eminent sense if the goal is to explain ‘discontent’ in a very broad sense. As suggested above, however, the resentment behind RWPP voting is quite specific, having to do with one’s own region (or country) falling behind other, richer regions (countries) because government (EU) policies favor the latter. We hypothesise that this type of resentment is most likely to develop in relatively equal regions that fall behind. Put differently, we hypothesise that vertical inequalities (class and ethnic/racial conflicts) distract from the populist framing of territorial grievances.

The second of our subsidiary hypotheses pertains to absolute income growth, as distinct from relative income growth. There is widespread consensus among political scientists and political economists that economic growth favors incumbent governments and promotes satisfaction with the current political order or, in other words, reduces political discontent (e.g., Vasilopoulou & Talving, 2023). In addition, it seems highly plausible to suppose that economic growth mitigates the negative effects of rising territorial or vertical inequality for low – and middle-income households or regions (cf. Poltier et al., 2023). Very simply, our hypothesis in this regard is that the resentment generated by falling behind richer regions or countries will be more pronounced when regional GDP per capita stagnates (or declines) than when regional GDP per capita grows in a robust manner.

## 2. Data, measurements, and descriptives

The core hypotheses that we seek to test rest on the idea that we need to distinguish between core and peripheral EU members states in order to understand cross-regional variation in RWPP support. Our operationalisation of this distinction is straightforward. As we conceive them, the ‘EU core’ consists of the original six members states, the three states that joined in 1973 (Denmark, Ireland and the UK) and the three states that joined in 1995 (Austria, Finland and Sweden) while the ‘EU periphery’ consists of the three Southern European states that joined in the early 1980s and the eleven East European states that have joined since 2004. As shown in Table 1, every one of the core member states has a GDP per capita that is significantly higher than the GDP per capita of the richest of the peripheral member states (Spain). Indeed, every core member state but one (Italy) has a GDP per capita that is higher than the GDP per capita of the European Union as a whole. It is also noteworthy that all the core member states have been stable democracies since the end of the Second World War while the peripheral member states are all relatively recent democracies (dating from the 1970s or 1990s).^10^

**Table 1.** The EU core and periphery distinction.

	2021 GDP/capita(current US$)	year of EU accession
*Core:*		
IE	100,172	1973
DK	63,007	1973
SE	61,029	1995
NL	57,768	1958
FI	53,655	1995
AT	53,638	1995
BE	51,247	1958
DE	51,204	1958
UK	46,510	1973
FR	43,659	1958
**EU**	**38,411**	
IT	35,658	1958
*Periphery:*		
ES	30,104	1986
SL	29,291	2004
ET	27,944	2004
CZ	26,821	2004
PT	25,568	1986
LI	23,723	2004
SK	21,392	2004
LA	21,148	2004
GR	20,193	1981
HU	18,728	2004
PL	18,000	2004
CR	17,568	2013
RO	14,858	2007
BL	12,222	2007

Source for GDP per capita: World Bank.

**Figure 1.**
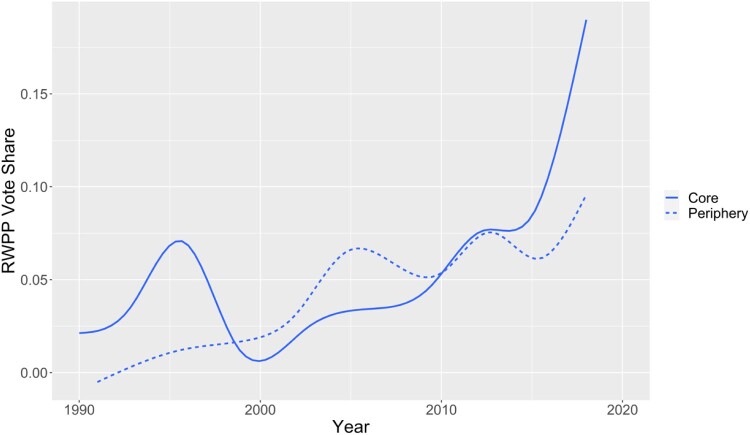
Smoothed time trends in RWPP vote share in core and peripheral EU member states. Note: Own graph, data taken from EU-NED.

**Figure 2.**
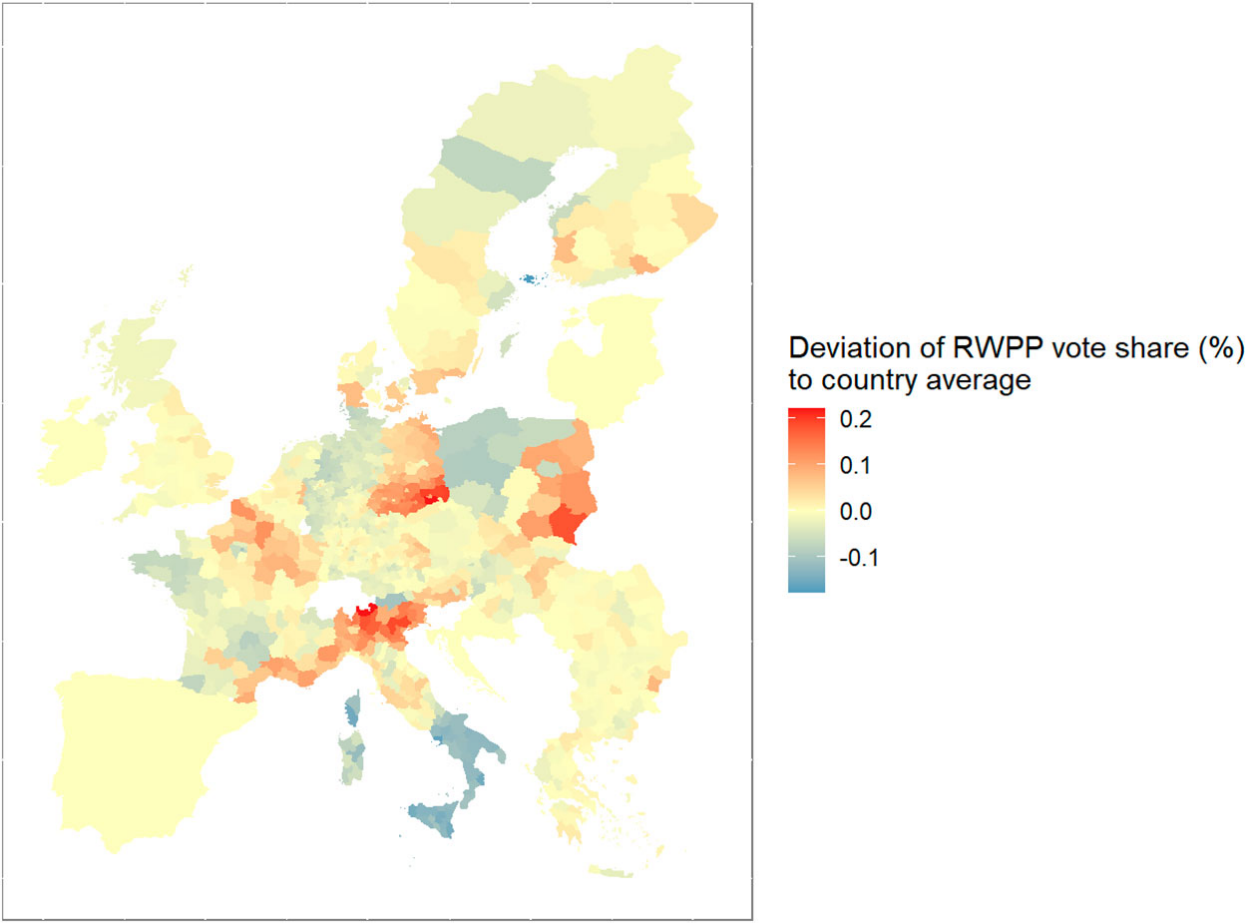
Regional RWPP vote shares in the second half of the 2010s, standardised around mean vote shares by country. Note: Own graph, data taken from EU-NED.

Separating the core and peripheral member states, Figure 1 displays time trends in the RWPP vote share as reported in the EU-NED dataset. The main take-away is that electoral support for RWPPs has increased steadily in peripheral member states since the early 1990s and that it has increased dramatically in core member states since the financial the crisis of 2007-2008. For the end of the period covered by EU-NED, Figure 2 in turn displays regional deviations from the average RWPP vote share of each country. The extent of regional variation in the RWPP vote share varies across European countries, but RWPP voting is geographically structured in most countries.

**Figure 3.**
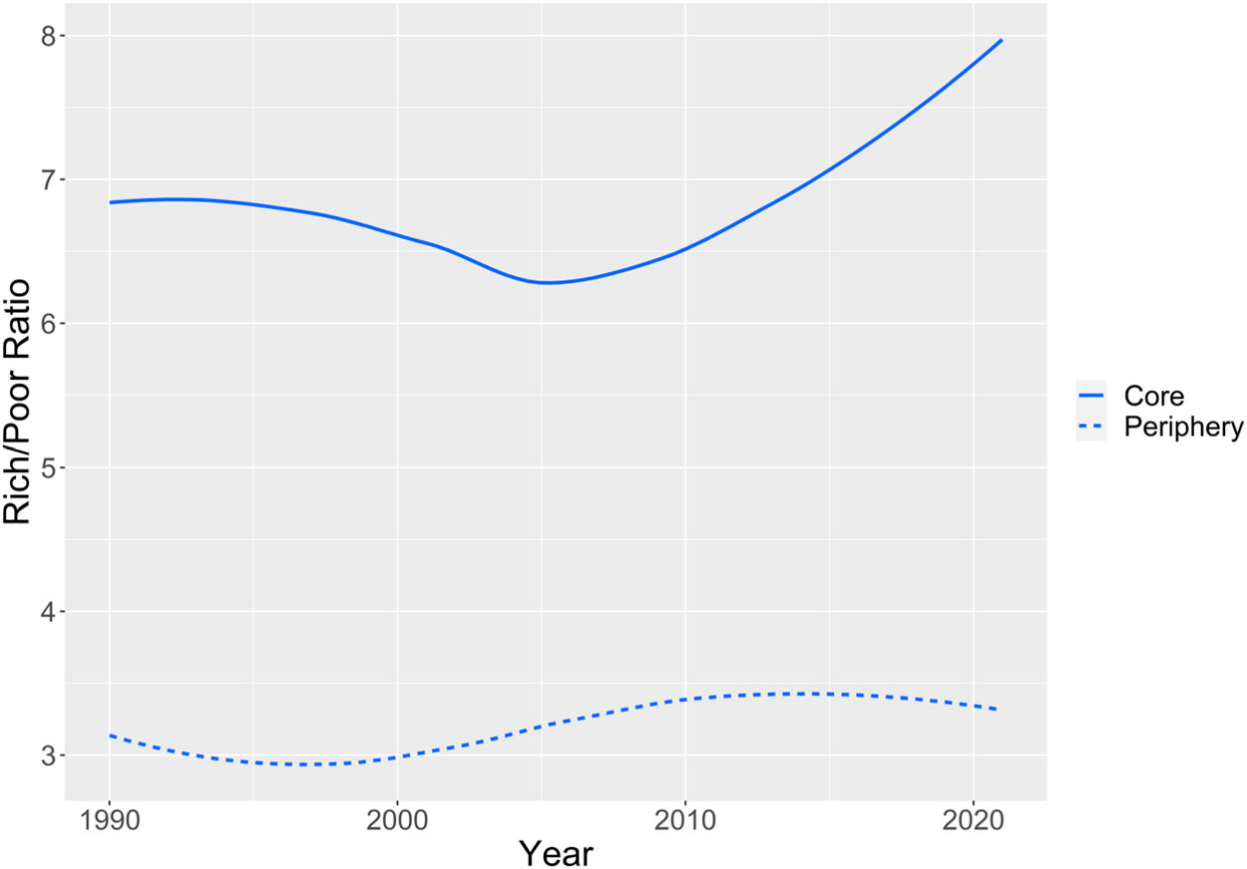
Time trends in richest-poorest ratios of GDP per capita (smoothed yearly cross-country averages).

Maintained by the European Commission, the ARDECO database includes estimates of GDP per capita for the regional units for which EU-NED provides data on party vote shares.^11^ With core and peripheral EU member states again separated, Figure 3 displays time trends in the average ratios of GDP/capita in the richest region to GDP/capita in the poorest region. By this measure, regional disparities are much more pronounced in the EU core than in the periphery. Moreover, we observe a significant increase of regional disparities in core countries, starting in the second half of the 2000s, apparently coincident with the rise of RWPPs. From 2006 to 2021, the richest-to-poorest ratio increased from 6.6 to 8.3 (an increase of nearly 26 per cent) in the EU core. By contrast, the richest-to-poorest ratio held essentially constant (around 3.4) in the EU periphery. As suggested above, mobilising resentment based on relative regional decline would appear to be much more of a ‘winning formula’ for RWPPs in the core than in the periphery.^12^

**Figure 4.**
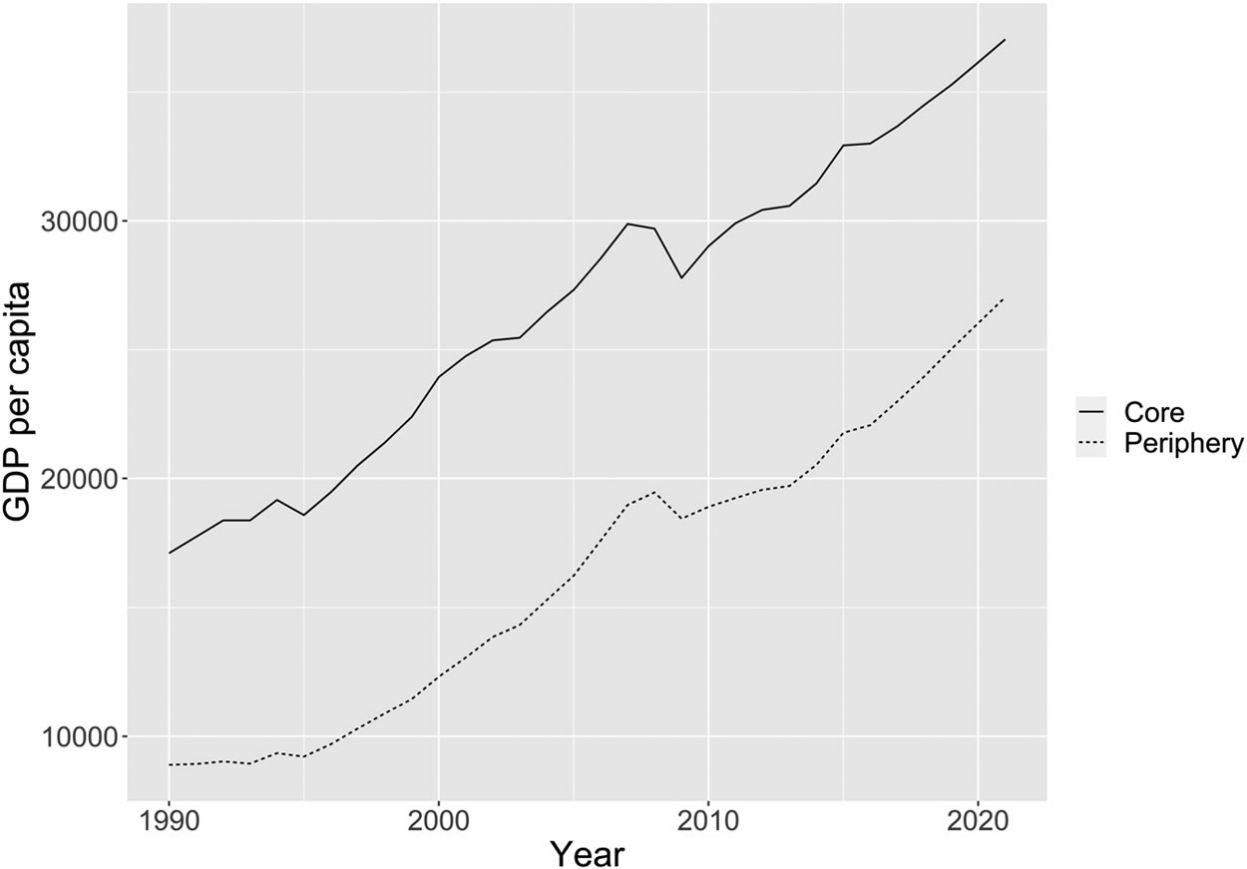
The GDP-per-capita gap between core and periphery over time.

Turning to cross-national income disparities, Figure 4 displays the evolution of average regional GDP per capita in the core and the periphery states since 1990. Average GDP per capita in the core was nearly 1.5 times higher than average GDP per capita in the periphery in 1990. By 2021, this ratio had increased to 2.2. At the same time, it is noteworthy that average GDP per capita has grown at a healthy pace in both groups of countries since the financial crisis, albeit at a slightly slower pace than in the 15 years preceding the crisis. On the face of it, Figure 4 would seem to call into question the idea that economic stagnation explains the rise of RWPPs in the 2010s.

As noted in the theoretical discussion, we are interested in exploring the question of whether (and how) income inequality within regions conditions the effects of territorial inequalities (falling behind the EU core as well as falling behind the richest region) on regional support for RWPPs. Again, our expectation is that the effect of falling behind will be more pronounced in regions that are relatively equal (homogenous) in a socio-economic sense. We do not have long time-series data of income inequality at the level of the regional units for which we observe RWPP vote shares (NUTS 2 regions for some countries, but NUTS 3 regions for most countries). However, the ARDECO database provides estimates of the manufacturing share of total employment for the same regional units. For the core EU member states, the manufacturing share of regional employment may plausibly serve as proxy for within-region inequality. Drawing on the work of Autor and collaborators (e.g., Autor & Dorn, 2013), the logic behind this idea is that routinised manufacturing jobs have historically provided relatively good earnings for semi-skilled and skilled workers located in the middle of the income distribution. As noted by Kurer (2020), among others, de-industrialization involves either a shift of employment into low-end services or a shift of employment into knowledge-intensive manufacturing and services. For our present purposes, the important point is that low-end as well as high-end post-industrial trajectories tend to be associated with a labor-market polarisation or, in other words, greater income inequality.

It is less obvious that the manufacturing share of regional employment can serve as a proxy for within-regional inequality in the EU periphery. Regarding the East European periphery, an extensive body of literature (notably Bohle & Greskovits, 2012) emphasise that foreign direct investment has been the key driver of industrial development since the collapse of state socialism and successful manufacturing has involved insertion into the global supply chains of West European multinationals (Ban & Adăscăliței, 2022). To the extent that this form of manufacturing depends on low wage costs, we would not expect regions with a strong manufacturing base to be characterised by a more egalitarian income distribution in the EU periphery.

**Figure 5.**
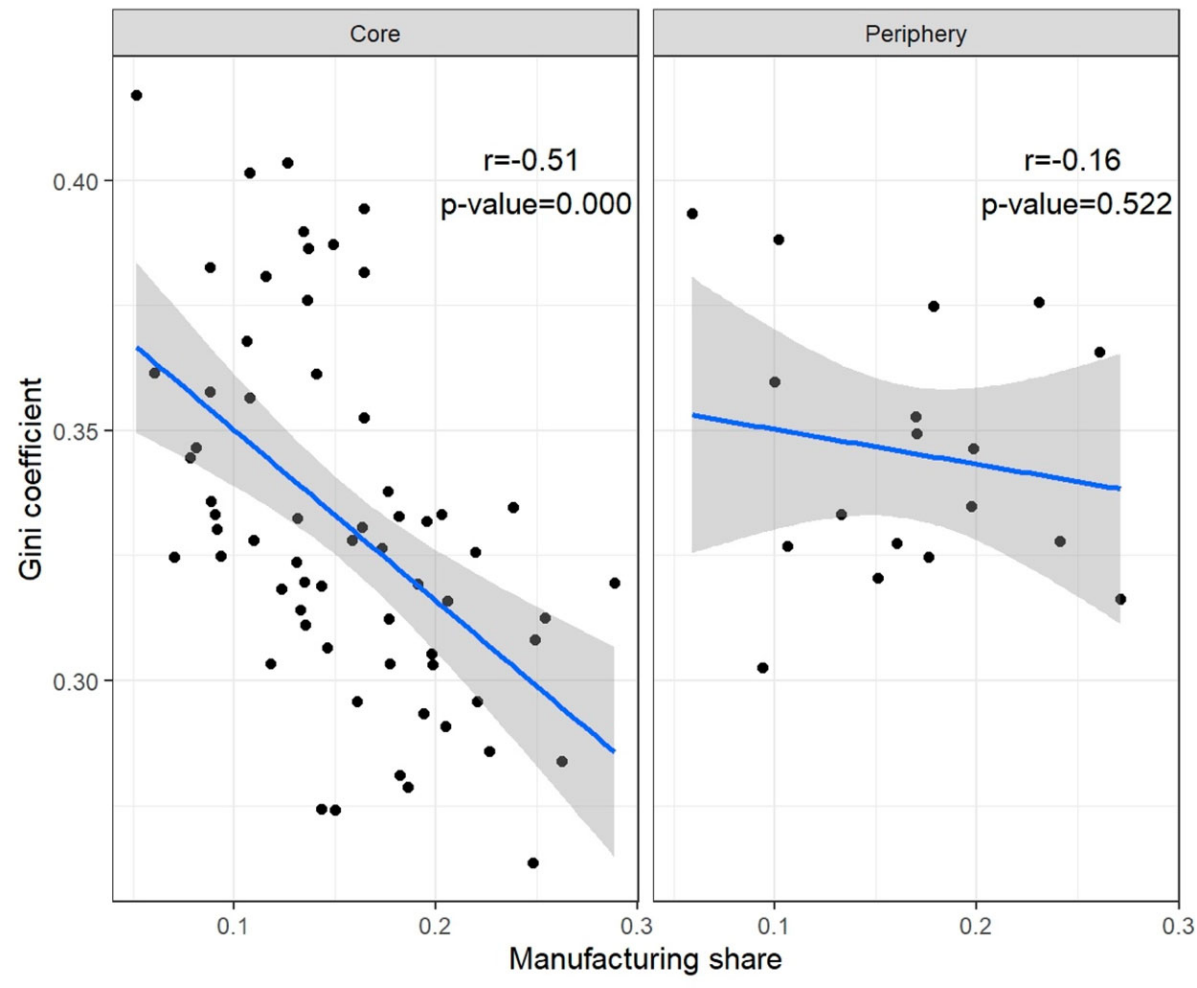
Regional Gini coefficients for disposable household income plotted against the manufacturing share of regional employment (averages for 2002–2009). Sources: https://dataverse.harvard.edu/dataset.xhtml?persistentId=doi:10.7910/DVN/RIOEAY and https://knowledge4policy.ec.europa.eu/territorial/ardeco-database_en.

Rueda and Stegmueller’s (2016) analysis of ‘externalities of inequality’ in Western Europe relies on estimates of within-region inequality (Gini coefficients for disposable household income) derived from the European Social Survey. For most countries, their estimates pertain to larger regional units than those in the EU-NED, but we can align our (ARDECO-based) data on manufacturing employment shares to their regional level for seven core EU member states (Austria, Belgium, Finland Germany, Netherlands, Sweden and the UK) and two peripheral EU states (Portugal and Spain). As shown in the left-hand panel of Figure 5, we do indeed find a strong correlation between the manufacturing share of regional employment and within-region inequality for core EU countries. By contrast, there appears to be no correlation whatsoever between manufacturing share and income inequality across the 18 peripheral regions from the Rueda and Stegmueller data (right-hand panel of Figure 5).

A great many studies invoke deindustrialisation to explain the rise of RWPPs and regional concentration of their electoral support (e.g., Broz et al., 2021). These studies typically posit that manufacturing workers are particularly vulnerable to globalisation as well as automation. At first sight, this might lead us to expect that populist support should be highest among former manufacturing workers (now unemployed or employed low-wage service jobs) and in regions that have undergone extensive deindustrialisation. As commonly noted, however, manufacturing workers and regions that rely heavily on manufacturing employment seem to be particularly attracted to RWPPs (Oesch & Rennwald, 2018). Articulated most forcefully by Kurer (2020), the standard solution to this apparent puzzle is that RWPP voters are motivated by fears about future income and status losses rather than ‘realized losses’ and that such fears are particularly pronounced among manufacturing workers (regions) in the context of economy-wide deindustrialisation (see also Im et al., 2022).

The argument that identifies fears of future losses as the key to RWPP support would lead us to expect the manufacturing share of regional employment to be associated with a higher RWPP vote share and perhaps also with a bigger positive effect of falling behind other regions or countries. The latter expectation is the same as our expectation based on the idea that manufacturing employment is a proxy for within-region inequality. The data that we analyze in this paper do not allow us to parse between these alternatives in any definitive fashion, but our own argument would seem to gain some credibility if it is the case that conditioning by manufacturing employment is stronger in the EU core than in the periphery.

## 3. Model specifications

The empirical results presented in the next section are based on estimating regression models with the regional vote share of RWPPs, ranging between 0 and 1, as the dependent variable. We estimate quasi-binomial generalised linear models (GLMs) to ensure that our model predictions remain within the boundaries of the dependent variable. We include year-fixed effects to account for trends and shocks that have affected all regional units and region-fixed effects to account for specificities that distinguish any given regional unit across all years. Moreover, we cluster standard errors over region and year (two-way clustering).^13^ Yielding estimates of how changes in our independent variables, pertaining to economic and demographic characteristics of regional units, affect changes in the regional RWPP vote share, our estimation strategy minimises concerns about spurious correlations and omitted-variable biases. That said, an obvious limitation of our meso-level analysis is that it does not speak directly to the mental (attitudinal) processes whereby relative regional performance affects voting behaviour.

In a first step, we estimate models with distance to the richest region of the country in which a region is located as the main independent variable of theoretical interest. As indicated above, we measure distance to the richest region as the ratio of the GDP/capita of richest region to the GDP/capita of the region for which we observe the RWPP vote share. This variable takes the value of 1 if the region in question is the richest region in the country in a given year and assumes increasingly positive values for poorer regions in the same year and country. The variable thus captures how well a given region, X, keeps up with economic growth in the most dynamic region of the country in which X is located. As the value of this variable increases, regions are falling behind the most economically thriving region of the country.

In a second step, we restrict our analysis to peripheral EU member states and estimate the effects of falling behind the EU core. For this purpose, the GDP/capita of the EU core is calculated as the population-weighted average GDP/capita of the eleven EU core member states. As with distance to the richest region in the same country, distance to the core is operationalised as the (year-specific) ratio of the GDP/capita of the core to the GDP/capita of the region for which we observe the RWPP vote share.

In each of these steps, the main models include regional GDP per capita as well as the manufacturing share of regional employment. In addition, we control for the regional employment and total population, conceived as additional indicators of socio-economic performance that might affect RWPP voting. Finally, we control for the economic strength of the agricultural sector within a given region, measured as the agricultural sector’s share at the gross value added. In so doing, we attempt to take account of the fact that farmers and other small business owners, along with manufacturing workers, appear to be particularly drawn to RWPPs (cf. Kitschelt, 2007).^14^ Temporally stable confounders, such as electoral rules or the geographical location of regions, are accounted for by region-fixed effects. Except for the two territorial inequality measures (distance to the richest and distance to the EU core), which already have meaningful units based on ratios, all variables are standardised; hence their effects should be interpreted as the effects of changes in standard deviations.

In a final step, we explore conditioning of the effects of falling behind by within-region inequality, proxied by the manufacturing share of regional employment, and by (absolute) changes in regional GDP per capita. We do so by estimating two-way interaction models separately for core and peripheral EU member states. Following Giesselmann and Schmidt-Catran (2022), we demean both of the variables that are interacted with each other to ensure that the estimated coefficients capture the joint effect of changes in the conditioning variable (manufacture share or GDP per capita) and changes in our measure of falling behind (distance to the richest region and distance to the EU core). Put differently, the data going into the calculation of interaction effects are exclusively based on within-regional variation. Again, we believe that a dynamic perspective, focusing on within-region changes, provides the most appropriate and convincing tests of our hypotheses about the effects of falling behind.

## 4. Empirical results

Table 2 presents our results for the effects of falling behind the richest region in the same country. Pooling all of our data, we find that falling behind the richest region is associated with an increase in the RWPP vote share (Models 2 and 3). We also find that increases in the manufacturing share of regional employment and the agricultural sector’s share of gross value-added are associated with increases in the RWPP vote share (Model 3). When we split the sample into core and peripheral EU member states (Models 4 and 5), the positive association between manufacturing employment and RWPP support only clears conventional thresholds of statistical significance for the sample restricted to peripheral member states. On the other hand, distance to the richest region and the agricultural sector’s share of gross value-added appear to be associated with increased RWPP support in the core, but not in the periphery. For the peripheral subsample, the coefficient for agriculture remains positive, but is nowhere near conventional thresholds of statistical significance, and the coefficient for distance to the richest region is actually negative (with *p* > .1).^15^ In view of the latter finding, it should come as no surprise that the coefficient for distance to the richest region becomes substantially bigger when we restrict the analysis to the EU core. For the EU core, our analysis suggests that – on average – a one standard deviation increase in the distance to the richest region is associated with a 3 per cent points increase in the RWPP vote share. Put differently, a region falling behind the richest area by one standard deviation, on average, responds with a 3 per cent points increase in RWPP voting.

**Table 2.** Quasi-binomial GLM of regional RWPP vote shares.

	(1)	(2)	(3)	(4)	(5)
				Core	Periphery
Distance to richest		1.019*** (0.2126)	1.207*** (0.2480)	1.377*** (0.2947)	−0.7034 (0.4190)
GDP pc	0.2206 (0.2482)		0.8181* (0.3744)	1.036* (0.3959)	−1.484* (0.6071)
Manufacturing share			0.5140** (0.1747)	0.3702. (0.1896)	0.4426* (0.1632)
GVA agricultural sector			0.3118* (0.1353)	0.3009* (0.1168)	0.0074 (0.2099)
Population			−0.7793 (0.7733)	−2.898** (0.9359)	0.8600 (0.8004)
Total employment			−0.2408 (0.2716)	0.9699 (0.6345)	0.5322* (0.2389)
Region FE	Yes	Yes	Yes	Yes	Yes
Year FE	Yes	Yes	Yes	Yes	Yes
S.E.: Clustered	two-way	two-way	two-way	two-way	two-way
Observations	7,471	7,471	7,471	5,768	1,703

Significance codes: 0 ‘***’ 0.001 ‘**’ 0.01 ‘*’ 0.05 ‘.’ 0.1 ‘ ‘ 1.

The fully specified model estimated with all data yields a significant positive coefficient for GDP per capita, implying that GDP growth, not stagnation, is associated with more support for RWPPs. We hasten to point out that there is no association whatsoever between GDP per capita and RWPP vote shares in a simple bivariate model (Model 1 in Table 2). Splitting the sample, the coefficient for GDP per capita is positive for the core and negative for the periphery (with *p* < .05 in both cases). Given that our measure of distance to the richest area is based on GDP per capita, we regard GDP per capita as control variable in these models, not really intended for substantive interpretation, yet it seems safe to conclude that, contrary to conventional wisdom, economic stagnation has not been a key driver of RWPP support in core EU member states.

As we have argued above, there are reasons to suppose that RWPP supporters in the peripheral EU member states are motivated by resentment of the wealthy EU core rather than richer regions in their own country (and that RWPPs in the periphery will focus attention on the EU core as a benchmark). Restricting the analysis to peripheral EU member states, Table 3 presents the results that we obtain when we estimate models that include distance to the EU core alongside distance to the richest region in the country to which a given region belongs. We do indeed find a significant effect of regional trajectories relative to the EU core: RWPPs have done significantly better in the peripheral regions that have fallen behind the EU core than in peripheral regions that have grown apace with the EU core even if these regions were, and remain, poorer than the poorest regions of the core. On average, falling behind the EU core by one standard deviation is associated with a 0.7 per cent points increase of the regional RWPP vote share. It is also noteworthy that introducing distance to the core does not change the non-significant effect of distance to the richest region. Our results suggest that RWPP voters in the periphery cue only on the EU core.

**Table 3.** Quasi-binomial GLM estimates of RWPP vote shares, peripheral countries only.

	(6)	(7)	(8)
Distance core	0.9493* (0.3626)	0.9875** (0.3240)	0.7970** (0.2361)
Distance richest		−0.6186. (0.3050)	−0.6259. (0.3193)
Manufacturing share			0.4979** (0.1703)
GDP pc			−0.6017. (0.3514)
GVA agricultural sector			−0.0359 (0.2067)
Population			−0.0751 (0.4809)
Total employment			0.2371 (0.1638)
Region FE	Yes	Yes	Yes
Year FE	Yes	Yes	Yes
S.E.: Clustered	two-way	two-way	two-way
Observations	1,703	1,703	1,703

Significance codes: 0 ‘***’ 0.001 ‘**’ 0.01 ‘*’ 0.05 ‘.’ 0.1 ‘ ‘ 1.

The effects of changes in the manufacturing share of regional employment are positive for both subsamples, but it only clears conventional significance thresholds for peripheral subsample. (In Table 2, the *p*-value of the coefficient for manufacturing clears the 90 per cent threshold when the fully specified model is estimated with data for core member states only). Contrary to what some of the literature on this topic seems to suppose, people who live in regions that are losing manufacturing jobs are not particularly attracted to RWPPs. To the extent that manufacturing employment matters, it is rather the case that people who live in regions that are retaining manufacturing jobs (becoming more manufacturing-reliant) are attracted to RWPPs.

**Figure 6.**
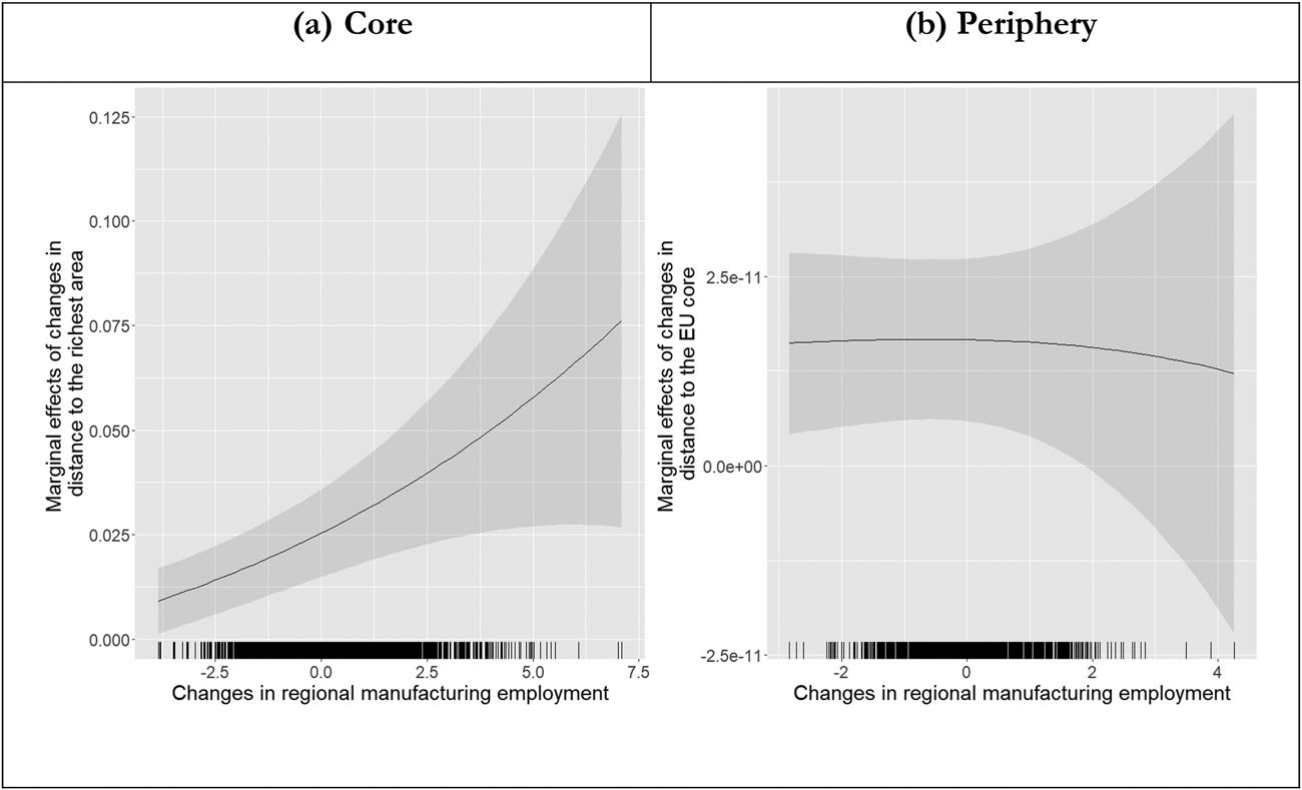
Effect of falling behind conditional on changes in manufacturing employment.

**Figure 7.**
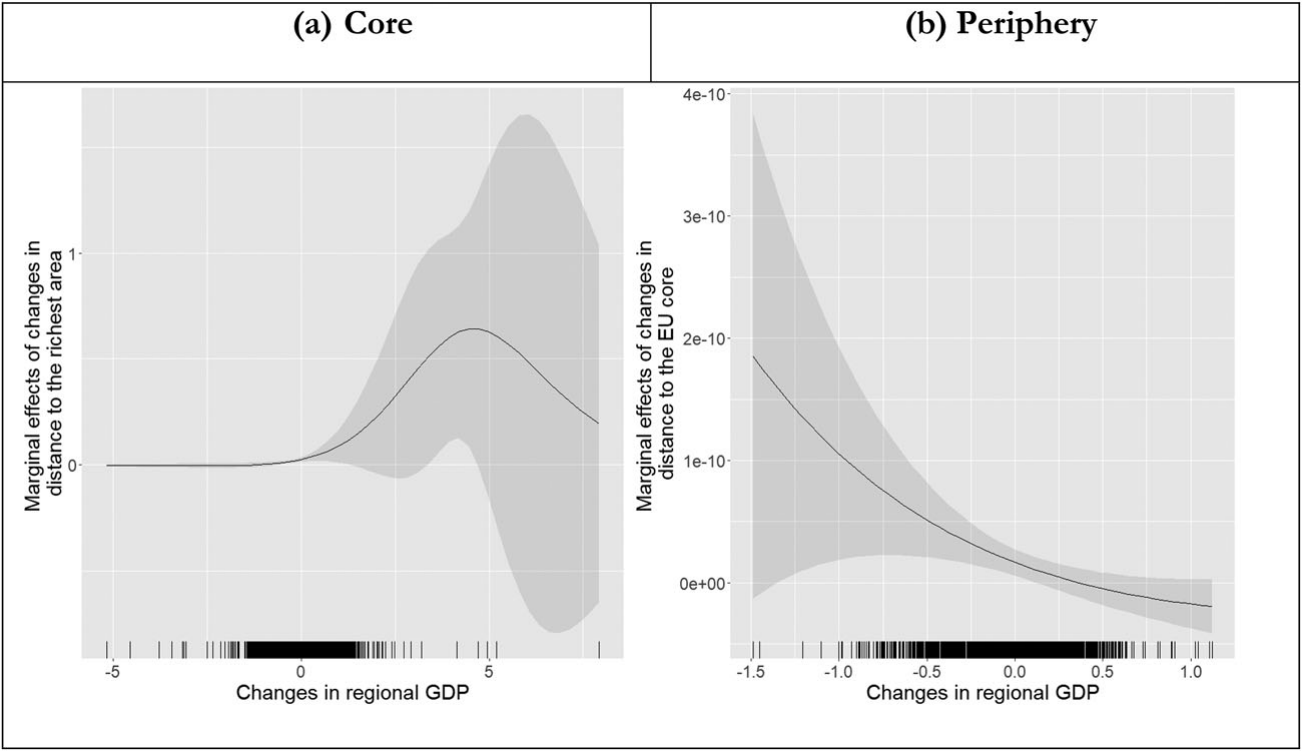
Effect of falling behind conditional on GDP growth.

We now turn to interaction models to explore how within-region inequality and absolute income growth condition the effects of falling behind. Table 4 presents the results of estimating these models separately for core and peripheral EU member states. For each subsample, we interact the most relevant benchmark – distance to the richest for the core subsample and distance to the core for the periphery subsample – with changes in within-region inequality, proxied by the manufacturing sharing of regional employment, and changes in regional GDP per capita. To facilitate the interpretation of these results, Figure 6 plots the effects on falling behind conditional on changes in the manufacturing share of regional employment while Figure 7 plots the effects of falling behind conditional on changes in regional GDP per capita.^16^

**Table 4.** Quasi-binomial GLM estimates of RWPP vote shares, with interaction terms.

	(9)	**(**10)
	Core	Periphery
Distance richest	0.5677*** (0.1151)	−0.6156* (0.2854)
Distance core		0.1371** (0.0388)
Manufacturing share	0.0641 (0.0515)	0.1224* (0.0451)
GDP pc	0.7569* (0.3126)	−0.5264. (0.2965)
GVA agricultural sector	0.2415* (0.1042)	0.0406 (0.1777)
Population	−2.824** (0.9072)	−0.2329 (0.4080)
Total employment	1.078. (0.6061)	0.1218 (0.1318)
Distance core*manufacturing share		−0.0182 (0.0205)
Distance core*GPD pc		−0.3765** (0.1291)
Distance richest*manufacturing share	0.0808** (0.0226)	
Distance richest*GDP pc	0.4672. (0.2421)	
Region FE	Yes	Yes
Year FE	Yes	Yes
S.E.: Clustered	two-way	two-way
Observations	5,768	1,703

Signif. codes: 0 ‘***’ 0.001 ‘**’ 0.01 ‘*’ 0.05 ‘.’ 0.1 ‘ ‘ 1.

As shown in Figure 6, changes in the manufacturing share of regional employment strongly condition the effects of falling behind the richest region in the core EU member states. According to these results, falling behind the richest region is only marginally associated with change in RWPP vote share when the manufacturing share of regional employment declines by 2.5 standard deviations from the mean. By contrast, a one-standard deviation increase in distance to the richest region is associated with around 1.25 per cent higher RWPP vote share when the manufacturing share grows by 2.5 standard deviations. For the EU periphery, we find that increased reliance on manufacturing employment has a direct positive effect on the RWPP vote share, but it does not condition the effect of falling behind the EU core (see right-hand panel Figure 6). The results for the core subsample can be read, we think, as evidence in support of the proposition that people in more equal regions are more likely (than people in less equal regions) to rally behind right-wing populist rhetoric and programmes when their region falls behind. We do not interpret the results for the periphery subsample as a refutation of this proposition. As suggested above, our preferred explanation of the divergence between the results for the subsamples is that manufacturing employment is a better proxy for within-region inequality in the EU core than in the EU periphery.

Looking at the results in Table 4, absolute changes in regional income growth (GDP per capita) appear to condition the effects of falling behind the EU core in peripheral member states, but not the effects of falling behind the richest region in core member states. For each subsample, Figure 7 presents marginal effects of falling behind on RWPP voting across changes in regional GDP per capita. In the EU core (left-hand panel), the marginal effects of falling behind the richest region are indistinguishable from zero across the entire range of changes in regional GDP per capita.^17^ For the periphery subsample, the interaction between falling behind the EU core and changes in regional GDP is statistically significant and the direction of this effects is consistent with the expectation that the effects of falling behind are amplified when regional incomes stagnate or decline. As Figure 7 illustrates, however, the estimated marginal effects are very small indeed. Even in the EU periphery, the conditioning effects of absolute income trajectories would appear to be substantively irrelevant. That said, it is important to keep in mind that our distinction between core and periphery is based on GDP per capita. According to our results, growth of GDP per capita does not condition the effects of relative economic performance on regional support for RWPPs, but levels of GDP per capita shape benchmarks for relative economic performance.

In the Supplementary Materials, we report on two robustness tests. First, we add two additional regional-level control variables: voter turnout and the vote share of incumbent parties (defined as parties with ministerial representation in the national government going into the election). While changes in the relative position of regions might have an impact on electoral participation and electoral participation might in turn affect the electoral fortunes of RWPPs, we control for incumbent vote share to ensure the observed effect of falling behind the richest region is not simply an anti-incumbency effect. As shown in Appendix 2, the substantive findings presented in Tables 2–3 are not affected by the addition of these variables.

Second, the dichotomous classification of countries as core or periphery represents another potential source of concern about the results presented above. There may well be important differences in the economic geography of RWPP support among core EU members and, conversely, among peripheral member states. As a step in this direction, Appendix 3 in the Supplementary Materials replicates results presented in Table 2 with Italy dropped from the subsample of core EU member states, on account of Italy having the lowest GDP per capita of the core member states (see Table 1). Again, the substantive findings presented in Table 2 are robust to omitting Italy from the analysis.

## 5. Conclusion

To summarise, the preceding analysis brings to light sharp contrasts between the economic geographies of right-wing populism in core and peripheral EU member states. In the core member states, falling behind the richest region in the country provides a favorable context for RWPPs to mobilise electoral support. In the peripheral member states, by contrast, within-country social comparisons seem to play a little role, but the wealth gap vis-à-vis the EU core provides another source of resentment that allows RWPPs to mobilise electoral support. In addition, our analysis indicates that RWPP support is associated with regional reliance on manufacturing employment in the periphery but less so in the core and that it is associated with economic stagnation in the periphery but not in the core. Finally, the effects of relative economic decline appear to be conditioned by changes in the manufacturing share of regional employment in the core but not in the periphery. In the core, the positive association between relative decline and RWPP support is much weaker in regions that are undergoing de-industrialization than in regions that are not undergoing de-industrialization.

Our interpretation of the interaction effect between relative economic decline and changes in the manufacturing share of regional employment posits that the manufacturing share is a good (inverse) proxy for within-region inequality in the core EU and that people living in regions characterised by less vertical inequality are more resentful of falling behind richer regions. The argument that within-region inequality dampens the effects of between-region inequality ought to apply to the periphery as well as the core, but we have reasons to believe that the manufacturing share of regional employment is not such a good proxy for within-regional inequality in the periphery. Generating direct measures of income inequality at the level of smaller regional units is an objective that we intend to pursue in future research.

The theoretical discussion framing our analysis leaves open the question of the extent to which the rhetoric and programmatic positions adopted by RWPPs shape the geographies of RWPP voting. There are at least two possible interpretations of the empirical results that we have presented. One interpretation posits that successful RWPPs are strategic actors that seize on horizontal economic disparities resented by large groups of voters and feature these disparities in their rhetoric and programmes and thus politicise (render politically salient) latent cleavages or, in other words, focus attention on some reference groups (benchmarks) at the expense of others. The alternative interpretation is that all these parties engage in essentially the same rhetoric of resentment and anti-elitism, but the resentful voters to whom such rhetoric appeals are motivated by different social comparisons. To parse between these interpretations would require a systematic, in-depth analysis of the rhetoric and programmes of RWPPs in Western and Eastern Europe. For the time being, suffice it to say that our analysis suggests that objective conditions constrain the ability of RWPPs to frame the ‘problem of territorial inequality.’ The Sweden Democrats may oppose the transfer of policy decisions to the EU level that has occurred over the last 20 years, but they cannot credibly argue that this transfer has benefitted people in some other country at the expense of Swedes. By comparison, such claims have much more traction in the Southern and Eastern peripheries of the European Union.

Related to the ability of political actors to focus public attention on some benchmarks rather than others, there is the thorny issue of how much people can be expected to know about objective conditions, conceived in terms of vertical as well as horizontal inequalities (see Gimpelson & Treisman, 2018; as well as Arel-Bundock et al., 2021). In our view, the issue here is not whether subjective perceptions are a better predictor of policy preferences and political behaviour, but rather whether perceptions change in response to objective conditions. Following Kayser and Peress (2012), the role of regional and national news media in informing cross-regional as well as cross-national comparisons of economic fortunes arguably deserves our attention.

An important limitation of the analysis presented in this paper is that it compares rich EU member states characterised by growing regional disparities with poor EU member states that have fallen behind the rich member states and have not experienced the same process of regional differentiation in economic fortunes. Our argumentation and empirical evidence invite an obvious question: What happens if (when) falling behind the core is combined with growing within-country disparities? Arguably, this is a scenario in which RWPPs would have the most room to maneuver, choosing to frame societal problems either in terms of falling behind the EU core or falling behind the richest region. Mindful of the loss of statistical power that this would necessarily entails, we intend to explore the question of how RWPPs behave and perform under such circumstances by leveraging variation among countries in the EU periphery in terms of (1) changes in (within-country) regional inequality and (2) the degree to which they have fallen behind (or kept up with) the rich member states.

More broadly, the analysis presented in this paper invites further discussion of how the politics of horizontal (territorial) inequalities differ from the politics of vertical inequalities, conceived in terms of income groups, classes, gender or ethnic groups (cf. Beramendi, 2012). As we have shown, the period since the financial crisis has been characterised, in the EU core, by sharp increases in regional disparities and a relatively stable distribution of household income measured by top income shares as well as the Gini coefficient. By contrast, the 15–20 years prior to the crisis were characterised by sharp increases in income inequality between households, especially top-end inequality, and a relatively stable distribution of income between regions. The post-crisis constellation has clearly favored right-wing populism. It is perhaps less obvious that the pre-crisis constellation favored left-wing politics, but the 1990s was a good decade for mainstream Left parties and to the extent that these parties lost electoral support in the 2000s, they primarily lost it to Greens and radical Left parties. Against the backdrop of rising top-end income inequality, the financial crisis clearly created an opening for left-wing populism in the Southern periphery of the European Union, most notably Greece and Spain, but the window of opportunity for left-wing populist parties seems to have closed quite abruptly in the early 2010s.

There are many things that progressive parties and the European Union need to do to respond to the rise of right-wing populist sentiments and parties. Our analysis suggests that one key challenge – perhaps *the* key challenge – for these actors is to develop policies that not only compensate regions and countries that have fallen behind in the near-term, but also promote more territorially equitable economic growth in the long-term.

## Supplementary Material

Supplemental Material

## Notes

1 While SD’s national vote share increased by 2.5 points, its Stockholm vote share increased by less than one percentage point. Statistics Sweden, https://www.scb.se/en/finding-statistics/statistics-by-subject-area/democracy/general-elections/general-elections-results/.

2 Dropping the three smallest member states (Luxembourg, Cyprus and Malta) and losing some additional observations for lack of data on independent variables, our analysis is restricted to 1,053 regional units in 25 countries. Regional units are NUTS 3 regions for 19 countries and NUTS 2 regions for 6 countries (Belgium, Ireland, Netherlands, Poland, Slovenia and the UK). Countries that have joined the EU since 1990 enter the dataset the year they obtained the status of an ‘accession country.’ The EU-NED dataset and codebook are available at: https://dataverse.harvard.edu/file.xhtml?fileId=6157990&version=1.1

3 We consider ‘right-wing’ to be interchangeable with ‘far Right’ and ‘radical Right.’ While many recent studies posit common determinants of left-wing and right-wing populism, Gonthier (2023) as well as Burgoon et al. (2019) emphasise differences in the motivations of individuals who support left-wing and right-wing populist parties.

4 It goes without saying that there is an important (inter-)subjective component to social status, but social status should not be conflated with the conflicts over cultural values emphasised by Inglehart and Norris (2019). In our understanding, a ‘materialist account’ does not imply that ‘objective conditions’ suffice to explain support for right-wing populist parties. Notable contributions to the literature that link right-wing populism to divergence in regional economic trajectories include Hobolt (2016), McNamara (2017), Rodríguez-Pose (2018), Essletzbichler et al. (2018), Carreras et al. (2019), Djikstra et al. (2019), Schraff (2019) and Adler and Ansell (2020). See Chou et al. (2022) for a broader discussion of the ‘localist turn’ in the study of populism.

5 All parties that we identify as ‘right-wing populist’ are also coded by PopuList as ‘Eurosceptic.’ See Djikstra et al. (2019) for a useful discussion of the overlap between populism and Euroskepticism.

6 As documented by Hense and Schäfer (2022), perceptions of not having any political voice are closely associated with voting for RWPPs across European democracies. See Lipps and Schraff (2021) on the effects of regional inequality on trust in national political institutions and EU institutions.

7 Other studies that identify differences in public attitudes across the divide between rich and poor EU member states include De Vries (2018), Vasilopoulou and Talving (2020), and Mayne and Katsanidou (2022).

8 Analyzing party programmes presented in *Land* elections, León and Scantamburlo (2022) provide a fascinating case study of how the AfD balances efforts to mobilise regional grievances with appeals to German national identity.

9 Our argumentation also draws inspiration from Scase (1977). Building on Runciman (1966), Scase shows convincingly that Swedish manual workers are much more likely to compare themselves to (upper) middle-class individuals than their British counterparts. He attributes the difference between his two samples to the structure of national union movements.

10 Note that our conceptualisation of the core-periphery distinction is fundamentally economic and thus different from similar distinctions by students of European integration (e.g., Schimmelfennig, 2016). Like the empirical analysis that follows, Table 1 excludes the three smallest EU member states. Luxembourg clearly belongs in the EU core by virtue of its GDP per capita ($131,511 in 2021) as well as being a founding member of the EU. The UK features as a core EU member state because our analysis pertains to the period from 1990 to 2018. Note also that all core member states were richer than all peripheral member states already in 1990.

11 ARDECO estimates of regional GDP per capita take purchasing power into account. Source: https://knowledge4policy.ec.europa.eu/territorial/ardeco-database_en.

12 The rise of regional inequality stands in sharp contrast to stability of personal (‘vertical’) income inequality in the EU core since the financial crisis of 2007–2008: averaging across the ten countries, the top 10 per cent share of pre-tax national income increased by less than 1 per cent from 2006 to 2021 (https://wid.world/). The Swedish case illustrates divergent trends in vertical and horizontal inequalities as well as regional variation in right-wing populist support. As defined by Statistics Sweden, the percentage of adults ‘at risk of poverty’ was 14.2 per cent in metropolitan areas and 14.3 per cent in smaller towns and rural areas in 2010. By 2020, the figure for metropolitan areas had dropped to 11.8 per cent while the figure for smaller towns and rural areas had increased to 20.3 per cent (https://www.statistikdatabasen.scb.se/pxweb/en/ssd/START__LE__LE0101/). Over the same period, the overall Gini coefficient for post-tax national income declined from .31 to .29 (https://wid.world/).

13 We prefer a fixed effects specification for the region and year levels rather than random effects as standard error estimates are more conservative. Moreover, there currently are no readily available estimation procedures that allow quasi-binomial link functions in multi-level GLM, as well as a lack of tools to calculate clustered standard errors.

14 Our measures of the aforementioned control variables are also based on ARDECO data (https://knowledge4policy.ec.europa.eu/territorial/ardeco-database_en). See Appendix 1 for descriptive statistics on all variables included in our analysis.

15 Note that there are 11 core countries and 14 peripheral ones, and still the *N* of the core-country sample is 4,888 and the *N* of the periphery-country sample 2,588. This is due to the fact the data for richer countries covers a longer time period and that richer countries tend to have more regions (e.g., Germany has around 400 NUTS3 regions).

16 The interaction effects reported in Table 4 are estimated using standardised variables and a double-demeaned estimator (Giesselmann & Schmidt-Catran, 2022).

17 The non-linear patterns at high values of GDP growth in the left-hand panel of Figure 7 result from statistical extrapolation, with very few observations driving these results (see rug plot below the marginal effects).

## Appendices

### Appendix 1. Descriptive statistics.

**Table:**

Variable	N	Mean	Std. Dev.	Min	Pctl. 25	Pctl. 75	Max
RWPP vote share	7446	0.055	0.082	0	0	0.085	0.69
GDP pc	7446	21814	9711	3738	15265	26360	78989
Distance rich	7446	3.135	1.581	1	1.822	4.056	11.316
Distance core	7446	1.417	0.685	0.244	1.033	1.588	7.067
GVA agricultural sector	7446	179.054	236.914	0	37.781	238.382	3057.576
Manufacturing share	7446	0.202	0.09	0.019	0.135	0.258	0.756
Population	7446	445342	596586	19600	133416	477418	6445530
Total employment (in 1000s)	7446	196.555	278.947	5.657	57.413	203.58	3443.903

### Appendix 2. Additional control variables.

**Table:**

	(1)	(2)
Distance Richest	1.374*** (0.2655)	−0.6020* (0.2912)
Distance Core		0.6938* (0.2528)
GDP pc	0.8744** (0.3139)	−0.6939* (0.3090)
Manufacturing Share	0.3464. (0.1695)	0.4721** (0.1622)
GVA Agricultural Sector	0.2450* (0.1004)	−0.0737 (0.1940)
Population	−2.621** (0.8611)	−0.3454 (0.4379)
Total Employment	0.9290. (0.5396)	0.2343 (0.1428)
Turnout	0.4467** (0.1583)	0.2193 (0.1675)
Incumbency vote share	0.2109* (0.0965)	−0.1597 (0.0945)
Region FE	Yes	Yes
Year FE	Yes	Yes
S.E.: Clustered	twoway	twoway
Observations	5,768	1,703

Signif. codes: 0 ‘***’ 0.001 ‘**’ 0.01 ‘*’ 0.05 ‘.’ 0.1 ‘ ’ 1.

### Appendix 3. Dropping Italy from core EU subsample.

**Table:**

	(1)
Distance Richest	1.450*** (0.3239)
GDP pc	0.7906 (0.3951)
Manufacturing Share	0.4309* (0.1905)
GVA Agricultural Sector	0.4116** (0.1366)
Population	−4.177** (1.246)
Total Employment	2.061** (0.7086)
Region FE	Yes
Year FE	Yes
S.E.: Clustered	twoway
Observations	4,888
