# Supplementary material for: Falling behind whom? Economic geographies of right-wing populism in Europe
Source: J Eur Public Policy. 2023 Nov 9;31(6):1591–619. doi: 10.1080/13501763.2023.2278647 (PMC11104744; doi:10.1080/13501763.2023.2278647)
Supplement: Supplemental Material [file RJPP_A_2278647_SM3908.docx]

**Appendix**

**Falling behind whom? Economic Geographies of Right-Wing Populism in Europe**

Dominik Schraff & Jonas Pontusson

Journal of European Public Policy, 2023

**Appendix 1**

Descriptive statistics

| Variable | N | Mean | Std. Dev. | Min | Pctl. 25 | Pctl. 75 | Max |
| --- | --- | --- | --- | --- | --- | --- | --- |
| RWPP vote share | 7446 | 0.055 | 0.082 | 0 | 0 | 0.085 | 0.69 |
| GDP pc | 7446 | 21814 | 9711 | 3738 | 15265 | 26360 | 78989 |
| Distance rich | 7446 | 3.135 | 1.581 | 1 | 1.822 | 4.056 | 11.316 |
| Distance core | 7446 | 1.417 | 0.685 | 0.244 | 1.033 | 1.588 | 7.067 |
| GVA agricultural sector | 7446 | 179.054 | 236.914 | 0 | 37.781 | 238.382 | 3057.576 |
| Manufacturing share | 7446 | 0.202 | 0.09 | 0.019 | 0.135 | 0.258 | 0.756 |
| Population | 7446 | 445342 | 596586 | 19600 | 133416 | 477418 | 6445530 |
| Total employment  (in 1000s) | 7446 | 196.555 | 278.947 | 5.657 | 57.413 | 203.58 | 3443.903 |

**Appendix 2**

Additional control variables

|  | **(1)** | **(2)** |
| --- | --- | --- |
| Distance Richest | 1.374*** (0.2655) | -0.6020* (0.2912) |
| Distance Core |  | 0.6938* (0.2528) |
| GDP pc | 0.8744** (0.3139) | -0.6939* (0.3090) |
| Manufacturing Share | 0.3464. (0.1695) | 0.4721** (0.1622) |
| GVA Agricultural Sector | 0.2450* (0.1004) | -0.0737 (0.1940) |
| Population | -2.621** (0.8611) | -0.3454 (0.4379) |
| Total Employment | 0.9290. (0.5396) | 0.2343 (0.1428) |
| Turnout | 0.4467** (0.1583) | 0.2193 (0.1675) |
| Incumbency vote share | 0.2109* (0.0965) | -0.1597 (0.0945) |
| Region FE | Yes | Yes |
| Year FE | Yes | Yes |
| S.E.: Clustered | twoway | twoway |
| Observations | 5,768 | 1,703 |

Signif. codes: 0 '***' 0.001 '**' 0.01 '*' 0.05 '.' 0.1 ' ' 1

**Appendix 3**

Dropping Italy from core EU subsample

|  | **(1)** |
| --- | --- |
| Distance Richest | 1.450*** (0.3239) |
| GDP pc | 0.7906. (0.3951) |
| Manufacturing Share | 0.4309* (0.1905) |
| GVA Agricultural Sector | 0.4116** (0.1366) |
| Population | -4.177** (1.246) |
| Total Employment | 2.061** (0.7086) |
| Region FE | Yes |
| Year FE | Yes |
| S.E.: Clustered | twoway |
| Observations | 4,888 |
